# Lossless Decompression Accelerator for Embedded Processor with GUI

**DOI:** 10.3390/mi12020145

**Published:** 2021-01-31

**Authors:** Gwan Beom Hwang, Kwon Neung Cho, Chang Yeop Han, Hyun Woo Oh, Young Hyun Yoon, Seung Eun Lee

**Affiliations:** Department of Electronic Engineering, Seoul National University of Science and Technology, Seoul 01811, Korea; hwanggwanbeom@seoultech.ac.kr (G.B.H.); chokwonneung@seoultech.ac.kr (K.N.C.); hanchangyeop@seoultech.ac.kr (C.Y.H.); ohhyunwoo@seoultech.ac.kr (H.W.O.); yoonyounghyun@seoultech.ac.kr (Y.H.Y.)

**Keywords:** lossless compression, inflate algorithm, hardware accelerator, graphical user interface, embedded processor, system-on-chip

## Abstract

The development of the mobile industry brings about the demand for high-performance embedded systems in order to meet the requirement of user-centered application. Because of the limitation of memory resource, employing compressed data is efficient for an embedded system. However, the workload for data decompression causes an extreme bottleneck to the embedded processor. One of the ways to alleviate the bottleneck is to integrate a hardware accelerator along with the processor, constructing a system-on-chip (SoC) for the embedded system. In this paper, we propose a lossless decompression accelerator for an embedded processor, which supports LZ77 decompression and static Huffman decoding for an inflate algorithm. The accelerator is implemented on a field programmable gate array (FPGA) to verify the functional suitability and fabricated in a Samsung 65 nm complementary metal oxide semiconductor (CMOS) process. The performance of the accelerator is evaluated by the Canterbury corpus benchmark and achieved throughput up to 20.7 MB/s at 50 MHz system clock frequency.

## 1. Introduction

With the development of the mobile industry, the user-centered application market has increased significantly and, as a result, the embedded system has become high-performance to meet advanced users’ requirements [1]. In particular, the embedded system is equipped with a display in order to provide a visible environment to a user interface and attempts to achieve a high-performance graphical user interface (GUI) in the embedded system have appeared [2]. As a result, not only the connection between the embedded system and the user, but also the provision of various types of sources is extended through the GUI [3,4]. Traditionally, the most essential elements to provide a GUI are the advancement of the memory capacity and processing time to store the source. However, the embedded system with the limited memory area has an essential challenge of storing the image data for GUI in minimum condition. Most systems that utilize large amounts of data exploit various data compression algorithms to overcome this problem [5,6]. However, in the case of an embedded processor, the workload that is demanded for the data compression and decompression causes an extreme bottleneck [5].

The way to alleviate this problem is to integrate the hardware accelerator, which is strong on the specific workload, as a part of the peripheral [7,8]. The embedded processor requests the decompression operation which occurs in the process of decoding to the specially designed hardware accelerator. As a result, the processor has a benefit of containing the resource to execute other commands continuously. Through the advantage acquired by employing the accelerator, the embedded processor maintains the system frequency with high performance and reaches the low-power consumption design [9,10]. In addition, an optimized system design is possible because of the area benefit generated by utilizing compressed data. Several compression algorithms have been researched to apply a compression accelerator on the embedded system [1,11]. The lossy compression algorithm has been dominant owing to the relatively high compression ratio [8]. However, in the case of image compression for constructing a GUI, a lossless compression can achieve a high compression ratio, as similar as to the lossy compression, while maintaining image quality [12,13].

In a GUI, the elements that are artificially produced to deliver clear information to users such as figures, Arabic numerals, icons, and symbols account for a large proportion. Although the natural image such as photograph or landscape may have an infinite number of embedded palettes, depending on the environment and object, the artificially produced images tend to select specific colors in order to eliminate ambiguity. This means that the probability of repetitive character strings is increased. The property makes it possible to obtain a high compression ratio through the window look-ahead buffer conventionally utilized in lossless compression [14,15]. Therefore, a hardware accelerator for lossless decompression is essential in order to utilize the lossless compressed data in an embedded system where the image quality deterioration is not allowed.

In this paper, we propose a lossless decompression accelerator (LDA) optimized for an embedded system with a GUI. The LDA is designed based on the inflate algorithm, which is a decompression algorithm of deflate compression known to be used in gzip or portable network graphics (PNGs). The accelerator supports the static Huffman method and LZ77 decoding in parallel, considering the embedded system to reduce processing time and minimize memory capacity. The checksum module based on Adler32 is designed to enable extensible input as the zlib compressed data format. Moreover, the LDA receives commands with the memory-mapped I/O method from the embedded processor through the system bus and fetches compressed data through direct memory access (DMA) to reduce the burden of the main processor. The LDA fetches the zlib format data that are compressed with up to 32 k window buffer without a separating preprocess. The fetched data are decompressed with the six-stage pipeline to minimize bottleneck. Finally, the decompressed raw data are transferred to the pre-assigned address by burst write to avoid long wait times for memory access. The embedded system including the LDA is implemented on a field programmable gate array (FPGA) to verify the functional suitability and is fabricated with Samsung 65 nm complementary metal oxide semiconductor (CMOS) process. The performance of the accelerator is evaluated by the Canterbury corpus benchmark, which is mainly used to test lossless data compression algorithms.

The rest of this paper is organized as follows. In Section 2, we describe the deflate/inflate algorithm in detail. Section 3 discusses some related works for the lossless compression system. Section 4 explains the optimized architecture of the LDA for the embedded system. In Section 5, we present the implementation and system-on-chip realization of the embedded system with the LDA and analyze the experimental results. Finally, we give a conclusion in Section 6.

## 2. Inflate Algorithm

**Figure 1:**
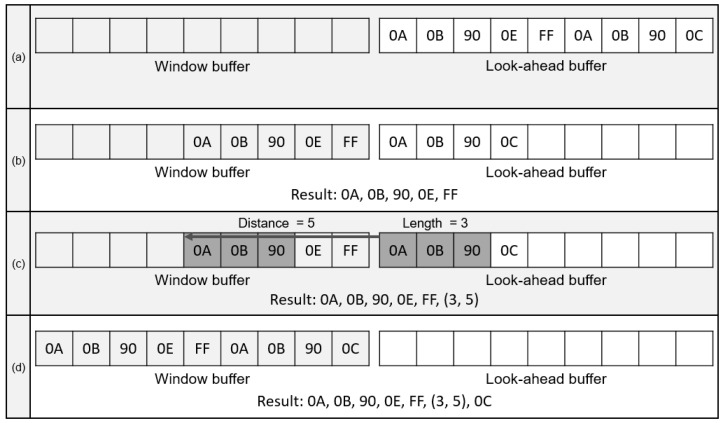
Process for LZ77. (**a**) String data to be decompressed; (**b**) Shifting data to Window buffer; (**c**) String data compression with distance and length; (**d**) Shifting duplicated string data.

As inflate algorithm is a decompression algorithm for deflate compression; back-ground knowledge of deflate algorithms is required. A deflate algorithm has processes for compressing repetitive string and assigning bits to the repetitive string in accordance with the frequency. For those processes, LZ77 and Huffman encoding are applied [16]. In LZ77 compression, a look-ahead buffer and window buffer are utilized. The data to be compressed are aligned in the look-ahead buffer and slide to the window buffer as the compression proceeds. The window buffer is scanned to find duplicated string starting on the front of the look-ahead buffer. Figure 1 shows the compression sequence of the LZ77 algorithm. In Figure 1a, string data from 0A to 0C are stored in the look-ahead buffer. As shown in Figure 1b, the character data are sequentially shifted to the window buffer until the duplicated strings are detected between the window buffer and the look-ahead buffer. In Figure 1c, the string data from 0A to 90 in the look-ahead buffer are duplicated with the string data in the window buffer. The length and distance of the duplicated string data are extracted as the length–distance pair, which is an output of the LZ77 compression [17]. Therefore, 0A to 90 characters are stored as (3,5), which refers to the number of duplicated character and distance. As LZ77 stores the duplicated string with two characters, LZ77 compression has an advantage when the compressed data are over three characters. When the length–distance pair is generated, the duplicated string and next character in the look-ahead buffer are shifted to the window buffer, as shown in Figure 1d. The length of buffers affects the compression ratio of LZ77 encoding because it represents the length of string data to be scanned. As the deflate format supports the window size up to 32 k-byte, a window buffer with identical size is required for optimal performance.

In deflate format, the LZ77 compressed data are additionally encoded with Huffman code. Huffman encoding is divided into static and dynamic encoding. Static Huffman encoding utilizes predefined code tables that allocate serial bits to the literal, length, and distance outputs of LZ77 compression, which are called symbols [16]. On the contrary, dynamic Huffman encoding constructs a Huffman tree according to the frequency of symbols [18]. By assigning a short-length code to the repeated symbols, dynamic Huffman encoding has an advantage of compression ratio [19]. As a result, static Huffman encoding shows a relatively low compression ratio compared with the dynamic Huffman encoding. On the other hand, less computation is required because the static Huffman encoding does not need to generate a Huffman tree.

In order to decompress deflate data, Huffman and LZ77 decoding need to be performed sequentially. A Huffman code is decoded by matching the compressed data with Huffman code tables in bit level. In the case of dynamic Huffman decoding, analyzing Huffman tree information that is included in the deflate header is required to construct the Huffman code tables. As the deflate algorithm use two Huffman code tables for the literal-length symbol and distance symbol, it needs to identified whether the matched Huffman code is the literal or length symbol by the predefined range of the symbols. When the decoded symbol is a literal, the literal itself is an output of inflate algorithm and shifted to the window buffer of LZ77. When the decoded symbol is a length, the distance to the next symbol to decode is determined due to the order of LZ77 compression outputs. Therefore, the decoded length–distance pair is utilized to extract the duplicated data.

## 3. Related Work

The deflate compression can be processed in parallel and the research for accelerating compression is performed [20,21,22,23]. In the case of decompression, there are several challenges for accelerating owing to the serial nature of deflate format [24]. The deflate data are compressed with the LZ77 compression and Huffman encoding. The deflate format includes multiple number of compressed data blocks and each block has a various length that continues until the end-of-block (EOB) code. When the type of the Huffman code is static, the length of the literal-length code and distance code are 7- to 9-bit and 5-bit long, respectively [7]. In dynamic Huffman code, the literal-length and distance code bits vary from 1 to 15 bits including extra bits. Moreover, the back-reference between the deflate blocks for decoding LZ77 implies the dependency of compressed data. Because of the various length of the deflate data block and Huffman code with data dependency, the inflate operation need to be processed serially, and it causes challenges for accelerating [18].

In order to address the limitation of decompression accelerating, the technique for parallel decoding has been researched [24,25,26,27]. Jang et al. [25] proposed speculative parallelization for deflate decompression. By identifying the boundaries of the deflate block by scanning the EOB code, each compressed block is decoded independently. However, the challenge of LZ77 decoding remains owing to the back-reference. Sitaridi et al. [26] proposed an alternate deflate format for massively parallel decompression on a graphics processing unit (GPU). The compressed data block includes starting offset values in the file header and the dependencies between the data blocks are eliminated in order to decode the Huffman and LZ77 compression in parallel. By applying the parallel decoding to both LZ77 and Huffman code, the authors achieved the increase of decompression throughputs over 13 GB/s on the GPU. Weißenberger et al. [27] presented a GPU-based parallel decoder for Huffman code, which is compatible with the original deflate data format. The authors utilized the self-synchronization property that the decoding process is synchronized after the block boundary. Yamamoto et al. [24] proposed a gap-array data structure, which is an array of the gaps of segments in the Huffman encoding process. The gap array is attached to the Huffman encoded data for accelerating Huffman decoding. Although the generation of the gap array requires additional operation in the Huffman encoding process, parallel decoding is enabled to achieve 1.26 to 2.63 times performance increase.

In [11,28,29,30], the designs of hardware accelerators for decompression were proposed. Koch et al. [11] presented hardware decompression techniques for embedded systems. The authors modified the compression algorithms such as run length compression, Lempel–Ziv, and Huffman encoding in order to apply the algorithms to hardware efficiently. They implemented the hardware accelerators of each algorithm on FPGA and compared the compression ratio. Lazaro et al. [28] designed a hardware decoder that applies dual core architecture in order to support static Huffman and LZ77 decoding. The decoder is implemented on FPGA with an embedded microprocessor. Satpathy et al. [29] presented a decompression accelerator that applies a dual-arithmetic logic unit (ALU) architecture and block-adaptive Huffman decoder. The dual-ALU is utilized to improve the serial bottleneck of Huffman decoding by matching the Huffman code with two additional arrays. Moreover, the block-adaptive technique, which skips a missing length code region, reduces wasteful computations, resulting in an additional 13% performance increase. The decoder was fabricated in 14 nm tri-gate CMOS. Ledwon et al. [30] designed the FPGA-based hardware accelerators for deflate compression and decompression using high-level synthesis (HLS). The decompressor supports static and dynamic Huffman decoding with the technique of joint length-distance decoding. The design achieved average throughputs of 196.61 MB/s and 97.40 MB/s at the static and dynamic decoding, respectively.

The contribution of this paper is that we propose a lossless decompression accelerator (LDA), which supports both the LZ77 decompression and static Huffman decoding in original deflate data format. The LDA is designed with Verilog hardware description language (HDL) and synthesized on both FPGA and Samsung 65 nm CMOS process. The techniques such as first-in, first-out (FIFO) control and DMA are applied to the design in order to optimize the design for the embedded system that has restrictions of area and performance. The throughput of the design is evaluated by Canterbury corpus benchmark in condition of system-on-chip (SoC) for embedded system. We constructed the SoC design by employing a Cortex-m0 processor with the LDA and achieved the throughput up to 20.7 MB/s. Moreover, we compared the design with another FPGA-based inflate accelerator in terms of area and throughput.

## 4. Lossless Decompression Accelerator

**Figure 2:**
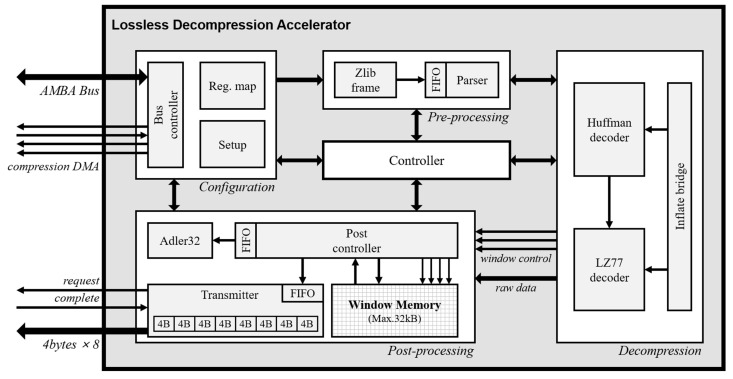
Block diagram of lossless decompression accelerator (LDA).

The decompression process consists of data import, Huffman decoding, LZ77 decoding, window buffer read/write, and data export. Data import and export operations are performed through memory devices with high-density storage such as synchronous dynamic random-access memory (RAM) in order to decrease the memory-area. Because the relatively slow memory speed and large data size make it hard to process with the embedded processor alone, the bus bottleneck is caused regardless of the decoding process. Besides, in the case wherein the decompression accelerator requires input data pre-processing, the main processor periodically implements the instructions to generate the input frame. The operating time for generating input frame is similar to the access time for memory. For this reason, the decompression accelerator must be an optimized design that minimizes the wait time by identifying the degree of bottleneck of each process in the target system. Figure 2 shows the block diagram of the LDA, which performs optimized operations for the embedded systems to reduce the workload of the main processor, as mentioned above. They are largely composed of four domains, and each domain is controlled through a main finite state machine in the controller. The description of each domain is as follows.

### 4.1. Configuration

The configuration domain supports the 32-bit system bus protocol that enables the interconnection with the main processor. The LDA includes seven registers that store control, error status, length, start address of compression data and raw data, and checksum result. Both big endian and little endian are supported and the LDA is configured with two input modes about the compression data, system bus, or direct memory access.

### 4.2. Pre-Processing

The data format structure that is compressed through the deflate algorithm has endian switch points because of the Huffman coding. For this reason, in the decoding process, the Huffman coding boundary must be detected and aligned by analyzing it in bit unit. As the LDA receives the data with 32-bit unit, the boundaries of the code appear randomly and the function for aligning them is required. This operation is performed in the pre-processing domain. First, the zlib frame module checks the header data of zlib format in order to identify the format validation. The data are transmitted to the parser module through the FIFO. The FIFO has 32-bit data width and 16 data depth in order to optimize area-time of the applied system. The parser module operates repetitive sort-flush function that performs shift operation by checking the length of processed bits according to the operation result of the decompression domain.

### 4.3. Decompression

In the decompression domain, the inflate algorithm is performed by receiving input data from the *parser* module with a nine-bit unit. The LDA always receives the compressed input data of static-Huffman code and extracts the symbol code only at one clock cycle through the pre-defined Huffman table. When the extracted symbol code is a literal code, the Huffman decoder module transmits the literal data to the post-processing domain. When the symbol code is a duplicated string, composed of the length and distance, the symbol code is transmitted to the LZ77 decoder module. The LZ77 decoder module sequentially extracts the length and distance through the symbol code and transmits decoding results to the post-processing domain. As the post-processing domain affects the memory bandwidth when the length–distance pair is transmitted, the LZ77 decoder module checks the status of post controller module in the post-processing domain before a result is transmitted.

The LDA performs the static-Huffman decoding to be optimized for the embedded system. In the case of dynamic-Huffman decoding, the decompression time is increased because additional workloads are required to create a Huffman tree. In order to analyze the efficiency of compression ratios for an artificially produced image, we compare the compression ratios of static and dynamic-Huffman encoding by employing the sample images, which have a size of 128 by 128. Table 1 shows the compression ratio results. The compression ratio is calculated as the following equation.

(1) Compression ratio (CR) = {1 − (compression data bytes/raw data bytes)} × 100(%)

As the produced image has a single background pixel characteristic in order to eliminate ambiguity, the difference in compression ratio is not different by even 1% owing to the LZ77 compression. As a result, the LDA gains a fast processing time and memory benefit through a slight decrease the compression ratio.

### 4.4. Post-Processing

The post-processing domain performs the window memory read/write operation according to the literal, length, and distance code of the previous domain and outputs raw data to the external memory in 32-byte unit. In this domain, the operations of Adler32 calculation, window memory access, and raw data transmission are executed in parallel. Therefore, the two FIFOs are embedded to minimize the waiting time by analyzing the workload for each operation. Because the range of distance value that affects the number of memory access is up to 32,768, the processing time is changed according to the length of the frame unit processed by the post controller module. We analyze the processing time according to the frame length for 1 byte and 4 bytes, which correspond to the literal symbol length and system bus width, respectively. The result is described in Section 5.

## 5. Realization

### 5.1. Chip Fabrication

**Figure 3:**
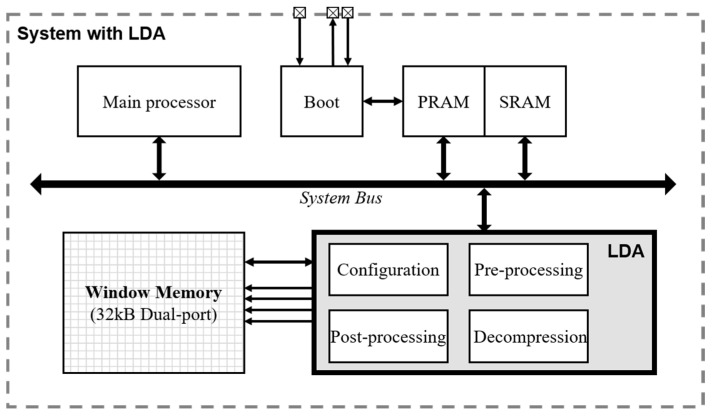
Block diagram of system-on-chip (SoC) with LDA. PRAM, phase-change random access memory: SRAM, static-random-access-memory modules.

Figure 3 shows the block diagram for the embedded system with the LDA. We utilize a Cortex-M0 processor as a main processor and the program data are stored in the phase-change random access memory (PRAM) through the boot module. The LDA is controlled by the instruction of the main processor via the system bus. The PRAM and static-random-access-memory modules (SRAM) of the SoC are single port memory. The window memory has 8-bit data width and 32 k depth with a dual port. The LDA directly reads and writes data to the window memory in accordance with the instructions of the main processor.

**Figure 4:**
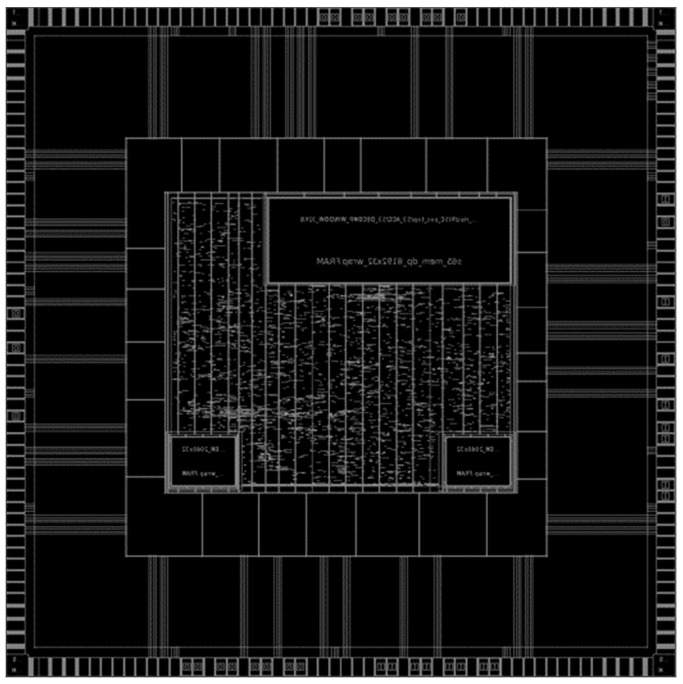
Layout of SoC with LDA.

We fabricated the SoC design as a Samsung 65 nm process. Figure 4 is a layout of the SoC design. The gate count of the proposed LDA is about 40.4 k based on the size of the two-input NAND gate in accordance with Synopsys Design Compiler. The SoC design is validated using Cadence NCsim and the layout is validated using Synopsys IC compiler and Cadence virtuoso.

### 5.2. Performance Analysis

In general, relatively slow memory is employed in the embedded system in order to achieve an area efficiency. Therefore, an optimized design considering the bottleneck of memory access is required. When the LDA accesses the window memory, reading and writing with 8-bit data width make it less difficult to process data because a literal output of deflate decompression has 1-byte data length. However, as the SoC design employs a 32-bit system bus, accessing the window memory with 4-byte data width affects the performance of the inflate operation. For this reason, we analyzed the throughput of the LDA according to the memory access width. Table 2 shows the difference of throughput between the access with 8-bit data width and the access with 32-bit data width. Compared with the throughput of 8-bit access, the throughput of 32-bit access is about 2.87 times higher on average and up to 3.39 times. Therefore, we applied 32-bit memory access to the LDA with the optimized method for window memory access.

We evaluated the performance of LDA with the Canterbury corpus benchmark, which includes several test files for lossless data compression algorithms. The functionality of the LDA was verified with the comparison of the raw data and original test files. Table 3 represents all the information of the Canterbury corpus test file and throughput of the LDA. The throughput was measured in the condition of the embedded system implemented with a Cortex-m0 processor and the LDA as explained above. As a result, the execution time for main processor and access time for window memory are included in the decompression time. In Table 3, the throughput was measured from 5.4 MB/s to 20.7 MB/s at a 50 MHz system clock frequency.

In order to compare our design with others, we implemented the SoC design on kintex7 Digilent genesys2 FPGA board and demonstrated the functionality of the LDA. The design utilizes the FPGA resource of 3362 lookup tables (LUTs) and 1950 flip-flops (FFs). In [11], the authors presented a deflate decompressor for FPGA-based embedded systems. The decompressor was implemented on several FPGAs such as Cyclone or Virtex-II with the resource utilization of about 5000 LUTs. Compared with the decompressor in [11], our design utilizes 33% less resources. In [7], the authors designed a deflate decompression accelerator using high-level synthesis, which supports both static and dynamic decoding. The design was implemented on Virtex UltraScale+ class FPGA with 10,736 LUTs and 6334 FFs. The throughput was evaluated with Calgary corpus benchmark and gained an average input throughput of 130.6 MB/s and output throughput of 386.6 MB/s in static decoding. The authors enhanced the design in [30] by adding joint length-distance decoding. As a result, they achieved input and output throughputs of 196.61 MB/s and 551.03 MB/s at 250 MHz clock frequency with 15,691 LUTs and 9122 FFs.

## 6. Conclusions

In this paper, we proposed a lossless decompression accelerator (LDA) for an embedded processor that supports LZ77 decompression and static Huffman decoding. We designed an SoC by employing a Cortex-m0 processor with the LDA. The design described with Verilog HDL and synthesized on both kintex7 FPGA and Samsung 65 nm CMOS process. The design utilizes 3362 LUTs and 1950 FFs of the FPGA resource and 40.4 k gate count based on the size of the two-input NAND gate. The resource utilization is less than that of other decompression accelerators and is efficient for an embedded system. The performance of the LDA is evaluated by the Canterbury corpus benchmark and achieved the throughput from 5.4 MB/s to 20.7 MB/s at a 50 MHz system clock frequency. A bottleneck is caused when the LDA accesses the window buffer because of the slow memory speed. We analyzed the delay of the decompression according to the access width of the window memory and alleviated the bottleneck with optimized post-processing.

## Figures and Tables

**Table 1:** Compression ratio between the dynamic-Huffman and the static-Huffman. CR, compression ratio.

No	Raw Data (Bytes)	CR of Dynamic (%)	CR of Static (%)	Gap (%)
1	65,664	92.2	91.7	0.52
2	16,512	88.5	88.2	0.25
3	16,512	95.0	94.4	0.61
4	16,512	94.9	94.3	0.54
5	16,512	93.6	93.0	0.55
6	16,512	94.8	94.2	0.53
7	16,512	94.1	93.4	0.67
8	16,512	88.6	88.1	0.66
9	16,512	95.2	94.5	0.71
10	16,512	92.0	90.9	1.18
11	65,664	93.8	93.4	0.44
Average	-	93.0	92.4	0.61

**Table 2:** Throughput between the 8-bit width access and the 32-bit width access.

No	Throughput of 8-Bits (MB/s)	Throughput of 32-Bits (MB/s)
1	10.9	31.7
2	12.0	21.6
3	12.2	37.4
4	12.2	36.9
5	12.2	38.3
6	12.2	36.8
7	12.1	33.2
8	11.9	40.4
9	12.2	38.0
10	12.0	28.4
11	11.2	32.6
Average	11.9	34.1

**Table 3:** Decompression performance on the Canterbury corpus benchmark.

File	Raw Data (Bytes)	CR (%)	Time (us)	Throughput (MB/s)
alice29	152,089	57.4	24,323	6.3
Asyoulik	125,179	52.7	22,217	5.6
Cp	24,604	62.2	3524	7.0
Fields	11,150	68.0	1341	8.3
Grammar	3721	61.1	543	6.9
Kennedy	1,029,744	71.9	108,447	9.5
lcet10	426,754	59.7	64,572	6.6
plrabn12	481,861	50.2	89,945	5.4
ptt5	513,216	87.6	24,797	20.7
Sum	38,240	63.2	5275	7.2
Xargs	4227	50.5	783	5.4
